# Viewing forests from below: fine root mass declines relative to leaf area in aging lodgepole pine stands

**DOI:** 10.1007/s00442-016-3621-6

**Published:** 2016-04-04

**Authors:** A. S. Schoonmaker, V. J. Lieffers, S. M. Landhäusser

**Affiliations:** Boreal Research Institute, Northern Alberta Institute of Technology, Peace River, Canada; Department of Renewable Resources, University of Alberta, Edmonton, Canada

**Keywords:** Chronosequence, Forest productivity, Forest decline, Root system development, Leaf area index

## Abstract

In the continued quest to explain the decline in productivity and vigor with aging forest stands, the most poorly studied area relates to root system change in time. This paper measures the wood production, root and leaf area (and mass) in a chronosequence of fire-origin lodgepole pine (*Pinus contorta* Loudon) stands consisting of four age classes (12, 21, 53, and ≥100 years), each replicated ~ five times. Wood productivity was greatest in the 53-year-old stands and then declined in the ≥100-year-old stands. Growth efficiency, the quantity of wood produced per unit leaf mass, steadily declined with age. Leaf mass and fine root mass plateaued between the 53- and ≥100-year-old stands, but leaf area index actually increased in the older stands. An increase in the leaf area index:fine root area ratio supports the idea that older stand are potentially limited by soil resources. Other factors contributing to slower growth in older stands might be lower soil temperatures and increased self-shading due to the clumped nature of crowns. Collectively, the proportionally greater reduction in fine roots in older stands might be the variable that predisposes these forests to be at a potentially greater risk of stress-induced mortality.

**Electronic supplementary material:**

The online version of this article (doi:10.1007/s00442-016-3621-6) contains supplementary material, which is available to authorized users.

## Introduction

It has been well documented that stand-level productivity peaks early on in forest stand development and declines slowly thereafter (Ryan et al. 1997; Smith and Resh 1999). Ryan et al. (1997) has provided the most comprehensive review of stand decline to date, and suggested a number of mechanisms for age-related decline, including: increasing hydraulic resistance with tree height [supported by Drake et al. (2010, 2011), although Ryan et al. (2006) disagree]; decreasing leaf area (e.g., Pearson et al. 1984; Smith and Resh 1999) either as a result of increasing crown abrasions (Rudnicki et al. 2003) as trees grow taller (Fish et al. 2006) or as a result of gaps in the canopy due to increased stem mortality in older stands (Xu et al. 2012; Binkley 2004; Sillett et al. 2010); increased respiration with age [partially supported by DeLucia et al. (2007), but more recently opposed by Drake et al. (2011)]; nutritional limitations in the soils of older stands [though this may not be that important according to Ryan et al. (1997)]; increased allocation to reproduction; and genetic changes related to meristematic age. Changes in stand structure (e.g., growth dominance or growth efficiency) have also been suggested as reasons for age-related decline (Binkley 2004; Tschieder et al. 2012; Binkley and Kashian 2015). Identifying and understanding the underlying factors driving this decline with stand age is crucial, as stress-induced forest mortality has been linked to mature forests (Allen et al. 2010) and there is increasing evidence that tree size (often driven by age) is correlated with increased mortality (Stahl et al. 2013, 2014; Nakagawa et al. 2000; Nepstad et al. 2007).

Proportional changes in the allocation of carbon to roots has also been noted as a potential factor in age-related declined (Ryan et al. 1997); this may be expressed as a total reduction in root mass or carbohydrate reserve storage in roots. However, root-based studies have received less attention due to the inherent difficulty in studying belowground structures and processes. Indeed, in a review of carbon allocation patterns in trees, Litton et al. (2007) stated that the “changes in flux and partitioning with forest development, particularly to belowground, remain poorly understood.” If there was a relative decline in fine root mass in older stands then it could be a factor in explaining decline in productivity, stomatal conductance and eventually leaf area. In a boreal lodgepole pine chronosequence, the allocation to leaves and branches remained relatively constant while stemwood production and root carbon allocation declined over time (Smith and Resh 1999). The results of Smith and Resh (1999) suggest that tree carbon partitioning favors leaf development over roots with age; if this is the case, the reduced allocation to roots should result in a negative feedback, impacting aboveground growth and leaf area development.

Many estimates of belowground carbon allocation are lumped into a parameter known as “total belowground carbon allocation” (Ryan et al. 2004; Litton et al. 2007). This approach does not separate roots by their function, i.e., the fine roots used in the acquisition of nutrients and water (analogous to aboveground leaf area) and the roots used for transport and structural support (analogous to the stem and branches). In addition, total belowground carbon allocation cannot be separated between that allocated to roots and mycorrhizae. Although larger suberized (woody) roots are capable of some water absorption, the rates observed are more than ten times lower than those of non-suberized fine roots (Gambetta et al. 2013). Root activity and growth are also soil temperature-driven processes; root activity might be affected by the tendency of older stands to have thicker insulating organic soil horizons resulting in lower soil temperatures (Minchin et al. 1994). Fine root responses might also be driven by the carbon reserves status of the roots and therefore be more sensitive to changing conditions, as these roots are the farthest organ from the site of carbon fixation (Landhäusser and Lieffers 2012) and are often associated with mycorrhizal fungi, which function as an additional carbon sink. However, root system development of both the fine and coarser root systems has never been directly measured and linked to stand leaf area development in a chronosequence study.

If chronosequence techniques are used to assess changes in stand dynamics over time, one key requirement will be that the sampled stands represent independent samples of the same common population. In addition, different stand ages should be matched in terms of site productivity, otherwise detection of age effects becomes difficult due to increased noise among stands. Incorporation of replication within stand ages ensures that the patterns observed between stand ages are not simply due to random variation among the stands vs. a true stand age effect. This paper explores the changes in fine root and leaf mass and area in a lodgepole pine (*Pinus contorta* Loudon) chronosequence across four age classes. We hypothesize that as stands age the fine root (<2 mm diameter) surface area and mass will decline more steeply than leaf area, though both are expected to increase to a point before an eventual decline. This would result in reduced capacity for soil resource extraction (water and perhaps nutrients) to supply the photosynthetic activity, thereby reducing carbon assimilation leading to declining wood volume production. In our holistic study we tracked the changes in the relationship between fine roots, leaf area and stem wood productivity as stands aged. This is the first replicated chronosequence study that concurrently measured all of these variables for forest stands.

## Materials and methods

### Site selection and characterization

The study sites were located along a 33-km north–south band, south of Hinton, Alberta (53°14.384′–117°28.596′ to 53°3.43′–117°4.145′). Elevation ranged from 1420 to 1577 m and all stands had south-facing aspects with slopes ranging from 3 to 33 % (Tables 1, 2). Soils were Dystric Brunisols and soil texture was primarily silty and sandy loamy and similar among sites. The elevation range is transitional between the upper foothills and the sub-alpine natural subregions with a C ecosite class (Beckingham et al. 1996). Understory plants common to all age classes included: *Vaccinium vitis*-*idaea, Linnaea borealis, Cornus canadensis, Elymus innovatu*s, and the feather mosses *Hylocomium splendens* and *Pleurozium schreberi*. Five replicate fire-origin stands were identified within each of the four age classes: 12 years—1997 fire, 21 years—1988 fire, 53 years—1956 fire, and ≥100 years—1910 fire (Table 1). Regional weather from a nearby station (1010-m elevation; coordinates 53.4°–117.54°) indicated that during the year of field measurements (2009), total precipitation was 429 mm and mean annual temperature was 3.6 °C (Environment Canada 2015).

**Table 1:** Geographic location, elevation, aspect and slope of study sites

Age class (years)	Site	Location				Elevation (m)	Aspect	Slope (%)	Year of fire	No. tree rings			
										Mean	SD	*n*	Site index
12	1	53°	10.676′	117°	28.810′	1564	SW	15	1997	8	1	16	12.5
12	2	53°	14.384′	117°	28.596′	1464	S	9	1997	8	2	16	12.5
12	3	53°	14.210′	117°	28.236′	1420	S	14	1997	9	2	15	13.5
12	4	53°	14.343′	117°	28.029′	1459	S-SE	23	1997	10	1	16	15.5
12	5	53°	14.344′	117°	27.740′	1502	S-SW	16	1997	9	1	15	14.0
21	1	53°	7.578′	117°	21.799′	1583	SW	12	1988	16	1	15	15.0
21	2	53°	7.237′	117°	21.379′	1499	S	18	1988	15	5	16	15.0
21	3	53°	7.195′	117°	22.849′	1521	S	3	1988	15	1	16	16.0
21	6	53°	7.379′	117°	21.432′	1577	S-SE	25	1988	16	1	16	16.0
53	1	53°	12.881′	117°	23.924′	1491	SE	9	1956	39	4	16	15.3
53	2	53°	12.708′	117°	24.007′	1449	S-SE	17	1956	37	5	16	15.3
53	3	53°	10.581′	117°	27.344′	1528	S	21	1956	37	5	15	15.3
53	4	53°	10.719′	117°	27.011′	1588	S	23	1956	38	6	16	16.0
53	5	53°	12.799′	117°	24.319′	1462	S	18	1956	45	12	15	14.5
≥100	1	53°	7.436′	117°	21.855′	1529	SW	33	1900–1910	93	15	16	13.5
≥100	2	53°	7.321′	117°	21.633′	1519	S-SE	24	1900–1910	88	11	15	15.3
≥100	3	53°	7.165′	117°	22.144′	1523	S	2	1900–1910	95	18	16	12.5
≥100	4	53°	4.891′	117°	9.154′	1472	SW	17	1900–1910	82	25	16	15.3
≥100	5	53°	3.430′	117°	4.145′	1455	W-SW	18	1900–1910	86	15	15	15.3

The known or estimated year of fire is also included as well as tree ring counts based on stem samples collected at stump height (0.3 m) for age classes 12 and 21 years and breast height (1.3 m) for age classes 53 and ≥100 years. Site index (at 50 years) was determined by the average number of tree rings at breast height and maximum tree height (Table 2) from site index curves of interior lodgepole pine (Farnden 1996)

**Table 2:** Tree and stand characteristics [height, diameter at breast height (or root collar diameter for 12- and 21-year age classes), live crown ratio and tree density, basal area, relative density (Curtis 1982) and stand density index (*SDI*) (Long 1985)] in 2009

Age class	Site	2009 Height (m)				2009 Diameter (cm)		Live crown length (m)		Live crown ratio		*n*	Tree density (stems ha^−1^)	Basal area (m^2^ ha^−1^)	Relative density	SDI
		Mean	SD	Max. 15 %	SD	Mean	SD	Mean	SD	Mean	SD					
12	1	1.0	0.5	1.9	0.2	1.7	0.8	1.01	0.49	1.00	0.00	90	31831	8.9	6.9	511
12	2	1.4	0.6	2.2	0.3	2.2	1.2	1.37	0.60	1.00	0.00	77	27233	13.8	9.5	701
12	3	1.5	0.7	2.5	0.3	1.9	1.1	1.46	0.72	1.00	0.00	118	41734	16.2	11.8	868
12	4	1.5	0.7	2.6	0.3	1.7	0.9	1.46	0.71	1.00	0.00	147	51991	15.7	12.0	884
12	5	1.2	0.6	2.3	0.4	1.5	0.8	1.23	0.58	1.00	0.00	172	60833	14.1	11.4	839
Age class 12		1.3	0.2	2.3	0.3	1.8	0.3	1.32	0.65	1.00	0.00		42724	13.7	10.3	761
21	1	2.1	0.9	3.5	0.4	3.6	1.6	1.82	0.87	0.81	0.13	97	19298	23.6	13.6	1006
21	2	2.3	1.1	4.0	0.2	4.2	2.6	1.98	1.14	0.82	0.09	56	11141	21.5	11.4	838
21	3	2.3	1.1	4.2	0.5	4.0	1.9	2.04	1.08	0.84	0.09	61	12136	18.4	10.2	751
21	6	3.7	1.0	4.8	0.1	5.5	2.2	2.88	1.09	0.77	0.17	62	12335	34.1	16.7	1234
Age class 21		2.6	0.7	4.1	0.5	4.3	0.8	2.33	1.15	0.78	0.14		13727	24.4	13.0	957
53	1	12.6	1.3	14.3	0.4	12.2	3.1	4.61	1.47	0.36	0.09	54	3508	43.4	15.8	1164
53	2	13.3	1.4	15.2	0.3	12.4	3.2	4.15	1.38	0.31	0.08	52	3378	43.2	15.6	1151
53	3	11.0	1.2	12.7	0.5	10.4	2.8	3.73	1.25	0.33	0.08	74	4807	43.6	16.9	1246
53	4	10.9	1.7	13.5	0.8	11.3	3.0	3.86	1.40	0.35	0.09	74	4807	51.2	19.2	1415
53	5	12.6	1.4	14.6	0.8	10.0	2.4	3.68	1.33	0.29	0.08	65	4222	35.2	13.8	1021
Age class 53		12.1	1.1	14.1	1.0	11.2	1.0	3.97	1.39	0.33	0.09		4145	43.3	16.2	1199
≥100	1	15.8	2.7	18.9	0.7	17.1	4.3	6.18	2.22	0.39	0.12	67	2133	52.2	16.5	1222
≥100	2	16.2	4.1	20.7	0.7	18.1	5.9	5.11	3.09	0.30	0.18	47	1496	42.4	13.1	964
≥100	3	14.6	2.4	18.0	0.8	16.3	4.1	5.30	1.94	0.36	0.12	70	2228	49.3	16.0	1178
≥100	4	16.0	2.9	20.1	0.7	17.8	5.0	6.05	1.99	0.37	0.08	54	1719	46.1	14.4	1061
≥100	5	17.3	2.8	20.6	0.5	17.4	4.1	6.37	1.85	0.37	0.08	62	1974	49.5	15.6	1153
Age class ≥100		16.0	0.9	19.7	1.2	17.3	0.7	5.82	2.25	0.36	0.12		1910	47.9	15.1	1116

Mean and maximum height (*Max. 15 *%) are shown with SD of the mean. Values were based on measurement of all trees within a circular plot. Note that plot size varied by age class: 12 year old—3.0-m radius, 21 year old—4.0-m radius, 53 year old—7.0-m radius, ≥100 year old—10.0-m radius

We selected stands of similar productivity and growth potential based upon a target site index of 15 [site index = height (meters) at age 50]; actual site index ranged from 12.5 to 16.0 m; as one stand from the 21-year age class exceeded this value it was subsequently removed. Site index curves for lodgepole pine (Farnden 1996) were used to estimate site index based on the average height of the tallest 15 % of trees within a plot. All stands in our study were considered fully occupied as they exceeded the minimum SDI value of 600 (Long 1985). Within each stand, we intended to sample an area containing approximately 70 trees plot^−1^, although the youngest age class tended to be oversampled at 77–172 trees plot^−1^. In the 12-year-old age class we measured 3-m-radius plots, 4-m-radius plots in the 21-year-old age class, 7-m-radius plots in the 53-year-old age class and 10-m-radius plots in the ≥100-year-old age class.

Within each sample plot in a stand, the total height, height at the base of the crown, diameter at 1.3-m height and stump diameter (30 cm from ground) were measured on all of the trees within the sample area described above. Two trees from each stand were destructively harvested for leaf mass and area and nitrogen concentration determination. Tree cross sections or increment cores were obtained from 16 trees from each stand for tree ring analyses.

To estimate soil nutrient availability in each site, five anion and five cation resin exchange probes (PRS™ Probes, Western Ag Innovations, SK) were installed at a 45° angle in the mineral soil (interfacing between the mineral A horizon and forest floor) and parallel to the base of the forest floor in the older age classes (above the mineral A horizon) in each stand in late May–early June and removed at 7.5 weeks (52 days) for analysis of soil macro- and micronutrients. Soil temperature at 10-cm depth was measured in each of the stands over the growing season using two Hobo^®^ temperature loggers (Onset, MA) programmed to record hourly soil temperatures.

### Fine root collection

In August 2009, one hundred and five randomly positioned locations were selected to sample soils from the upper 30 cm of the soil profile. Soil profiles cores were separated into two layers: 0–15 cm and 15–30 cm. Previous studies found that over 90 % of fine roots were located in the upper 20 cm [*Picea abies* (Ostonen et al. 2005)] or 30 cm [*Pinus sylvestris* (Xiao et al. 2003)] of the soil profile. In the current study, we also found that across all study sites, an average of 83 % (SD 10 %) of all the fine roots collected were in the upper 15 cm of the soil profile. In addition, at one site (100-year-old age class), root cores were sampled an additional 10 cm deeper (30- to 40-cm depth) in order to assess the relative quantity of roots missed at lower depths. At this location, 7.6 % (SD 7.4 %) of fine roots and 3.6 % of coarse roots were found at the 30- to 40-cm depth.

The area of soil from which roots were extracted ranged from 0.03 to 0.17 m^2^ subplot^−1^ depending on stand age (sample area—17 × 17 cm for 12- and 21-year-old stands, 34 × 34 cm for 53-year-old stands and 41 × 41 cm for ≥100-year-old stands). Five soil samples were collected in the 12- and 53-year-old stands and six samples in the 21- and ≥100-year-old stands. This amounted to sampling a total area of 0.145 m^2^ in each 12-year-old stand, 0.173 m^2^ in a 21-year-old stand, 0.578 m^2^ in a 53-year-old stand and 1.01 m^2^ in a ≥100-year-old stand, representing 0.3–0.5 % of the total stand area. Sampling was more intensive relative to other studies, e.g., Xiao et al. (2003) sampled 0.003 % of a 73-year-old *P. sylvestris* stand, Ruess et al. (1996) sampled 0.036 m^2^ per stand and Litton et al. (2003) 0.048 m^2^ for each stand (13 years old). Samples were collected in heavy-duty plastic bags and roots enclosed in soil and stored at 3 °C until processing (Ruess et al. 1996); refer also to Online Resource B for additional details on field sampling procedure.

### Root density determination

The root washing and separation procedure followed Teste et al. (2012) and is described in detail in Online Resource B. Washed fine roots were immersed in water and homogenized by cutting them into 4- to 6-cm fragments. A sub-sample (or complete sample when there were few roots) of fine roots was then removed and scanned on a flatbed scanner and surface area determined with image analysis software (WinRHIZO, Regent Instruments). All roots were oven-dried at 70 °C overnight or until weight constancy and then weighed. Fine root density (kilograms or square meters) was expressed relative to the size of the sampling area (square meters).

### Leaf area index

Given the wide range of crown structure across the age classes, we produced allometric relationships between tree age, diameter and height to estimate stand-level leaf area index (LAI) (detailed description in Online Resource C). This is because indirect measures such as those achieved by the LAI-2000, AccuPAR or SunScan all underestimate LAI compared with methods that utilize allometric scaling (Bréda 2003). Moreover, tree foliage in pine stands is clumpy and increases with age (Fish et al. 2006), therefore the error associated with these measures is not constant through stand development.

### Tree leaf nitrogen

The same samples utilized for determination of LAI were subsequently used for determining leaf nitrogen concentrations. Dried leaf samples were ground to pass a 40 mesh (0.4 mm) in a Wiley-Mill (Thomas Scientific, Swedesboro, NJ). Total nitrogen was determined using the Dumas combustion method (Sparks 1996) with a 4010 CHNS analyzer (Costech Analytical Technologies, Valencia, CA). Nitrogen concentration in needle tissues is presented as a percentage of total dry weight.

### Estimation of annual wood volume increment

Tree cores or cross sections were obtained from 16 trees (from a range of sizes) within each of the 19 stands. Cores and cross sections were taken at stump height (30 cm) for the 12- and 21-year-old stands and at breast height (1.3 m) for the 53- and ≥100-year-old stands. Cores and cross sections were oven-dried and sanded (400 grit) in order to identify annual rings. Tree cross sections and cores were scanned with a flatbed scanner and the number of rings and width (in two positions at a 90° angle) determined with image analysis software (WinDENDRO; Regent Instruments). When rings were difficult to see with the scanner, ring width was verified manually on a Velmex stage microchrometer and microscope. From the tree ring data, the diameter of measured trees in 2004 was calculated. These trees formed the basic data set from which linear models were developed within age classes to predict 2004 diameter and mean annual volume increment (Online Resource D).

### Data analysis

All analyses, including those described above, were carried out using R (R Core Team 2014). Parameters for which a single value was collected for each site, including leaf area, wood volume increment, leaf nitrogen, soil nutrients and soil temperature as a function of age, were analyzed as linear models.

Diagnostic plots of fitted vs. residuals were used to check for equal variances and histograms of residuals used for assessment of normality. The linear models function was used when assumptions of normality and equality of variance were met. However, when there was indication of unequal variance, the generalized least squares (GLS) function was used with a parameter to allow for unequal variances by age class. This was further supported by Akaike information criterion comparisons of the linear model with the GLS model (lower was better) (Anderson 2008). Root mass diameter classes were analyzed as linear mixed-effects models with a random effect for the site included (multiple soil cores collected per site). The function Linear mixed-effects models (R package NLME) was used when assumptions of normality and equality of variance were met. However, when there was indication of unequal variance, an additional parameter was added to allow for unequal variances by age class.

Graphical presentation of estimated means, confidence intervals and least-significant difference intervals (LSD) are shown in all subsequent figures. Graphical methods allow the reader to use their own judgment when meriting the statistical, or more importantly, the biological meaning of the data presented (Cohen 1994; Di Stefano 2004; Johnson 1999). All analyses were presented as means by age class with 95 % confidence intervals and LSD:

1 $${\text{LSD}} = \frac{\text{Model residual error}}{\sqrt n } \times \sqrt 2 \times t\,{\text{value}}$$

LSD were used as a visual method of multiple comparisons between age classes (Crawley 2007). Confidence intervals are presented in order to graphically depict the precision of the mean estimates (Cumming and Finch 2005).

## Results

### Edaphic factors

**Fig. 1:**
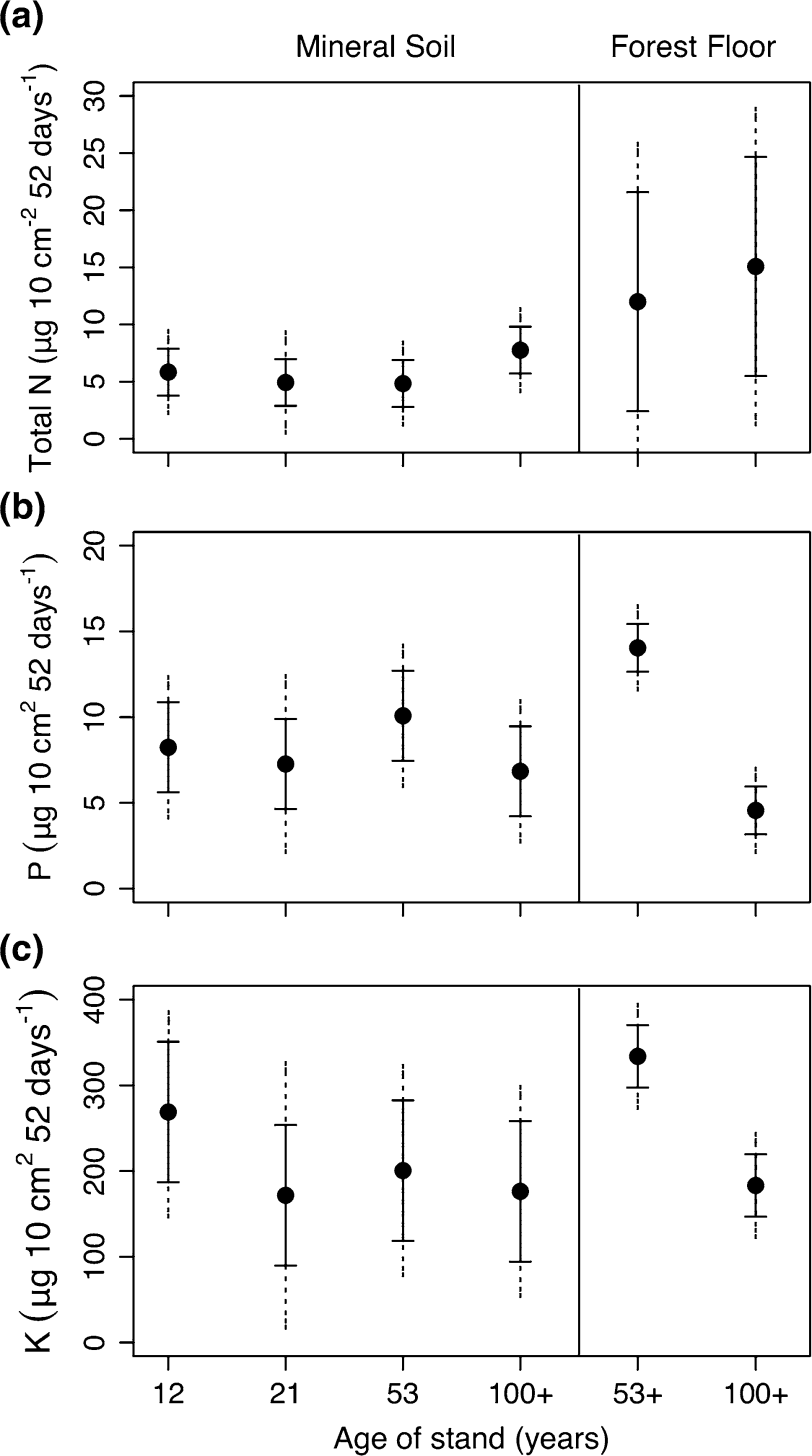
Soil available macronutrients recovered from Plant Root Simulator resin probes (five probes pooled in each stand into a single sample) averaged by age class (year of fire) for the period June–July 2009. **a** Total nitrogen (nitrate and ammonium), **b** phosphorus and **c** potassium. Means to the *left* of the *vertical line* were resin probes inserted at a 45° angle into the mineral soil. Means to the *right* of the* vertical line* were resin probes inserted horizontally at the forest floor-mineral soil interface. This could only be accomplished in the older age classes as the younger age classes lacked forest floor development. *Solid error bars* represent least significant difference intervals (LSD) and *grey dashed error bars* represent 95 % confidence intervals (CI) (*n* = 4–5)

**Fig. 2:**
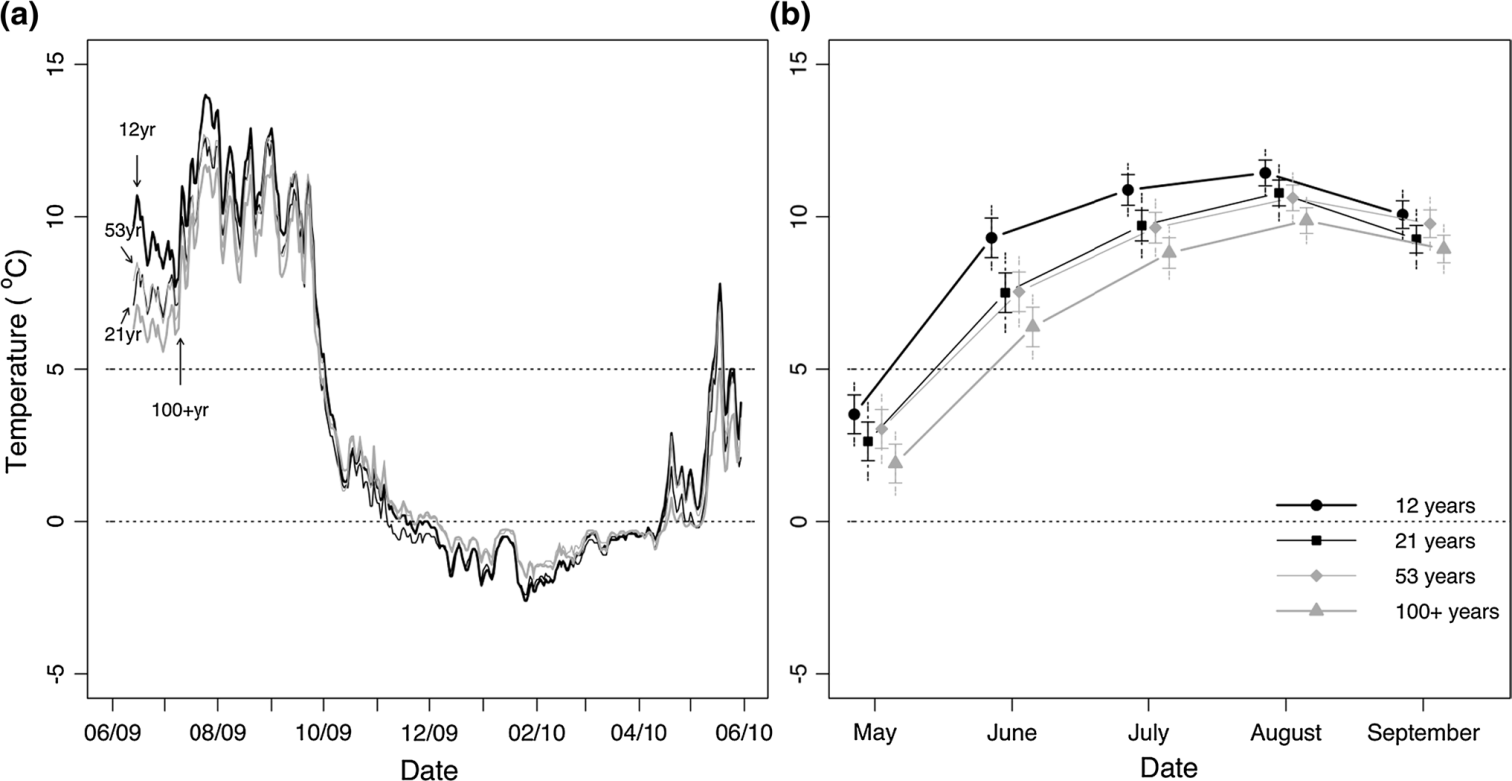
**a** Daily mean soil temperature (at 10-cm depth) for each of four stand age classes. Two temperature sensors were logged hourly at each stand; *lines* are means of all sensors within each stand age. **b** Mean monthly soil temperature for each of four stand age classes. *Solid error bars* represent LSD and *grey dashed error bars* represent 95 % CI (*n* = 3–5). *Dotted lines* indicate temperatures at 5 and 0 °C. For abbreviations, see Fig. 1

There was no difference in total available nitrogen across all stand ages, but the trend was for more available nitrogen in the oldest age class (Fig. 1a). Phosphorus and potassium were also similar across all age classes in the mineral soil but clearly declined between 53- and ≥100-year age classes in the forest floor interface (Fig. 1b, c). Soil temperatures during the growing season at 10-cm depth were lowest in the ≥100-year age class and highest in the 12-year age class (Fig. 2a). The difference between these stands was 1–3 °C on average (Fig. 2b). The trend was reversed in winter (Fig. 2a).

### Root development

**Fig. 3:**
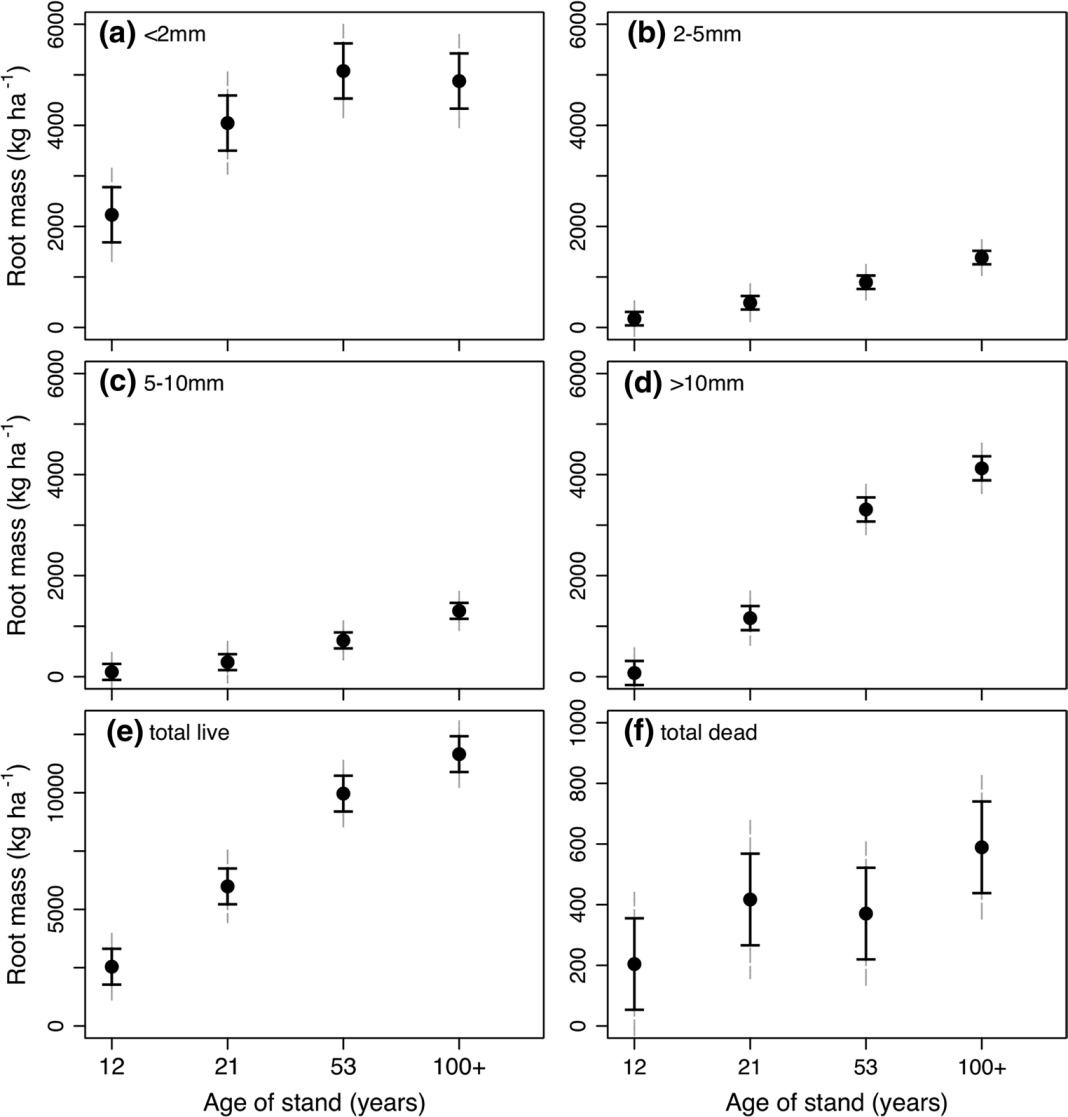
Stand-level root mass for root diameter classes **a** <2 mm, **b** 2–5 mm, **c** 5–10 mm, **d** >10 mm, **e** total live mass and **f** total dead root mass. Five to six soil cores were averaged for each stand and age class means represent four to five stands. *Solid error bars* represent LSD and *grey dashed error bars* represent 95 % CI. Note that *axis scale* is the same for **a**–**d** but different for **e**, **f**. For abbreviations, see Fig. 1

For the fine root diameter class <2 mm, there was a distinct increase in root mass from the 12- to 21-year-old and 21- to 53-year-old stands with no difference between 53- and 100-year-old stands (Fig. 3a). For all other diameter classes (2–5 mm, 5–10 mm and >10 mm) root mass consistently increased with age (Fig. 3b–d). Total root mass of all diameter classes increased steadily between age classes but more slowly between age 53 and 100 years (Fig. 3e). Total mass of dead roots (coarse and fine) nearly tripled from the 12-year-old stands to the 100-year-old stands (Fig. 3f).

**Fig. 4:**
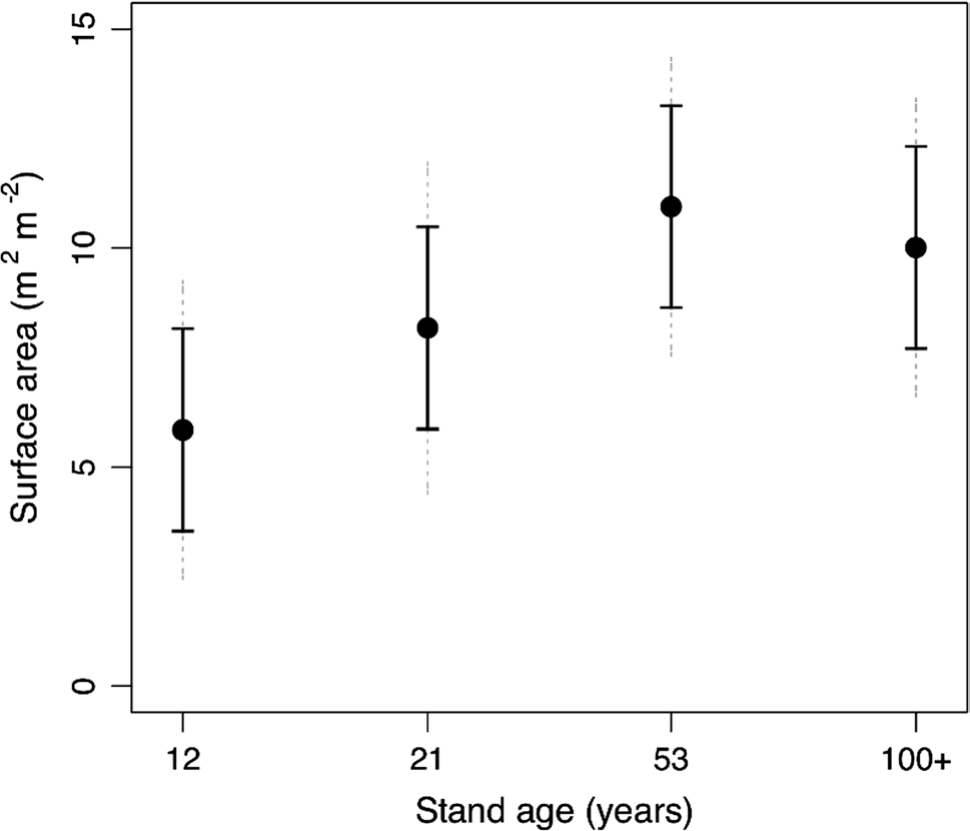
Stand-level root surface area of fine root diameter classes (<2 mm). Five to six soil cores were averaged for each stand and age class means represent four to five stands. *Solid error bars* represent LSD and *dotted error bars* represent 95 % CI. For abbreviations, see Fig. 1

Fine root area continued to increase with stand age up to 53 years and these parameters then stabilized between the 53- and ≥100-year age classes (Fig. 4). Root morphology also varied through the stand ages as specific root length (length/mass) and specific root area (area/mass) were highest in the youngest age classes (12 and 21 years) and lowest in the older age classes (53 and ≥100 years) (Table 3). The youngest stand, in particular, had nearly twice the root length per unit mass compared with the oldest age class (Table 3).

**Table 3:** Specific leaf area (*SLA*), fine root specific root length (*SRL*) and specific root area (*SRA*) of four lodgepole pine age classes with least significant difference intervals (*LSD*) and 95 % confidence intervals (*CI*)

Age class	SLA (cm^2^ g^−1^)			SRL (m g^−1^)			SRA (×10 cm^2^ g^−1^)		
	Mean	LSD	95 % CI	Mean	LSD	95 % CI	Mean	LSD	95 % CI
12	64.8	56.5–73.1	53.3–76.3	12.5	10.8–14.2	10.1–14.9	18.7	17.1–20.3	16.5–21.0
21	54.4	46.0–62.7	41.5–67.2	11.5	9.8–13.2	8.9–14.2	17	15.3–18.6	14.5–19.5
53	42.1	33.7–50.3	30.5–53.5	8.4	6.7–10.1	6.0–10.8	13.6	11.9–15.2	11.3–15.8
≥100	50.7	42.2–59.0	39.1–62.1	6.3	4.6–8.0	4.0–8.7	11.6	10.0–13.2	9.4–13.8

Values are averages from eight to ten trees within each age class for SLA. Root parameters are based on scanned, sub-sampled fine root measurements from each of five to six excavated pits per stand (*n* = 4–5 stands per age class)

### LAI and nitrogen concentration

**Fig. 5:**
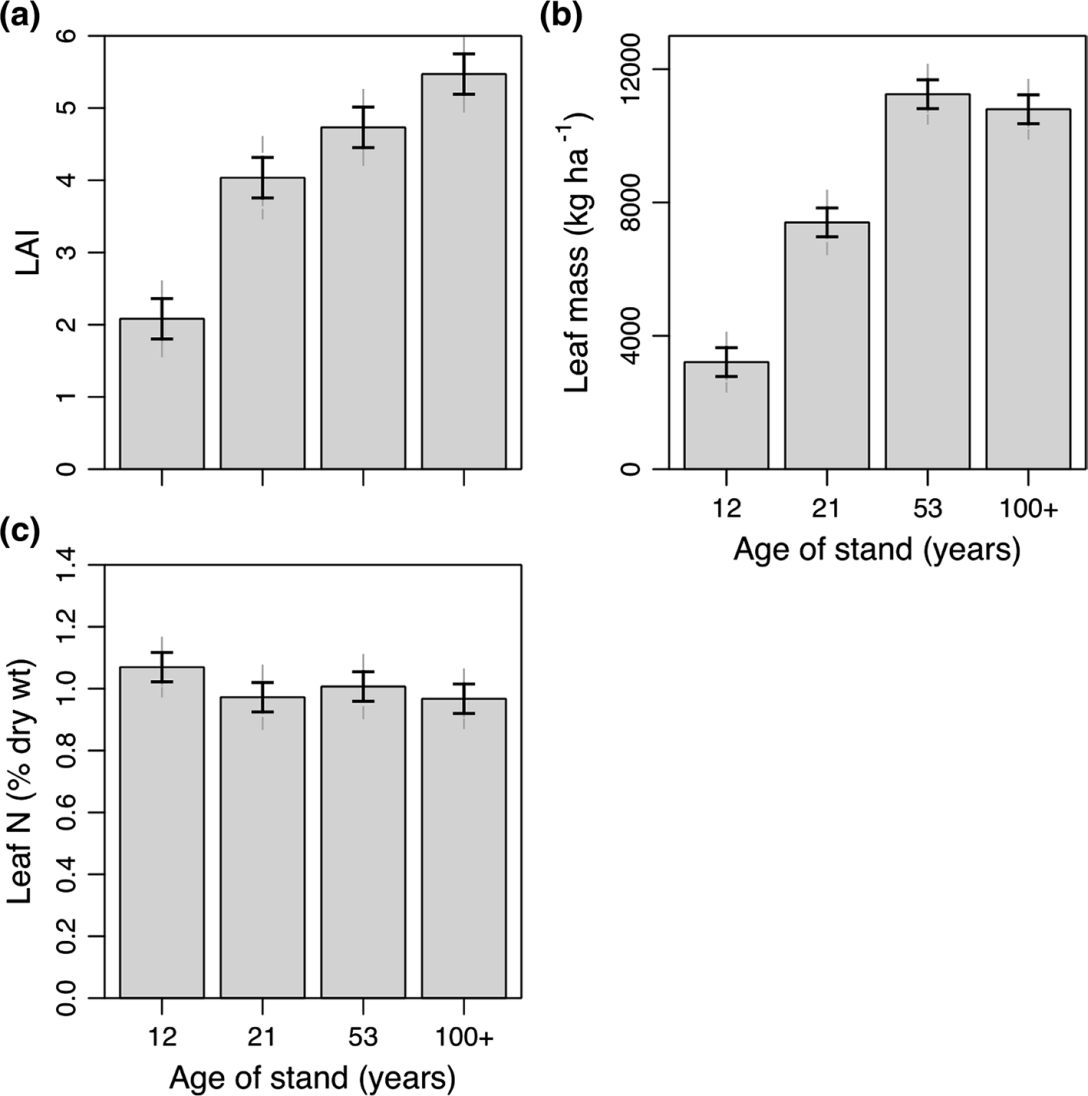
**a** Leaf area index (*LAI*) averaged by age class (year of fire) and **b** leaf mass (kg ha^−1^) by age class. **c** Leaf nitrogen concentration in August 2009. *Solid error bars* represent LSD and *grey dashed error bars* represent 95 % CI (*n* = 4–5). For other abbreviations, see Fig. 1

LAI nearly doubled from 2 to 4 between age 12 and age 21 years (Fig. 5) and continued to increase steadily across the older age classes, peaking at ~5.5 at age ≥100 years (Fig. 5a). However, when expressed as leaf mass (kilograms per hectare), the two oldest age classes showed similar values at ~11,000 kg ha^−1^ (Fig. 5b). Specific leaf area declined with age and was at its lowest (42.1 cm^2^ g^−1^) in the 53-year-old stand but then appeared to increase again to 50.7 cm^2^ g^−1^ in the oldest age class (Table 3). Leaf nitrogen concentration was highest in the youngest age class, at 1.1 % on average, and then stabilized to 1.0 % in subsequent age classes (Fig. 5c).

**Fig. 6:**
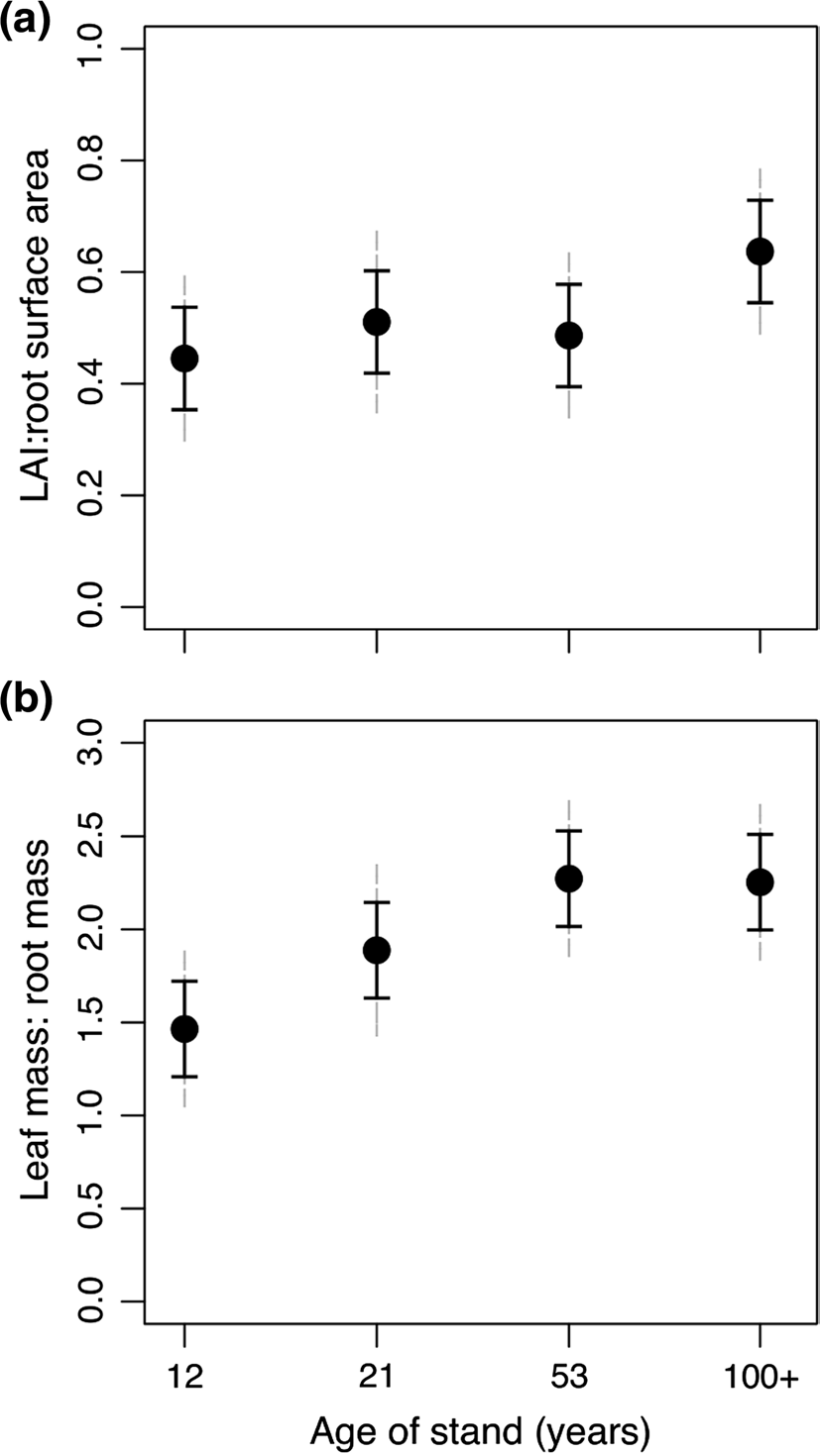
**a** LAI: root surface area ratio of fine roots (<2 mm) averaged by age class (year of fire). **b** Leaf mass:root mass ratio of fine roots (<2 mm) averaged by age class (year of fire). *Solid error bars* represent LSD and *grey dashed error bars* represent 95 % CI (*n* = 4–5). For other abbreviations, see Figs. 1 and 5

Age-driven changes in leaf and root morphology impacted the balance between LAI and root area with stand age. The ratio of LAI:fine root surface area was constant between the 12- and 53-year age classes and increased in the ≥100-year-old age class (Fig. 6a). When expressed on a mass basis, the ratio tended to increase between 12- and 53-year-old stands and then plateaued from 53- to ≥100-year-old stands (Fig. 6b).

### Wood volume increment and growth efficiency

**Fig. 7:**
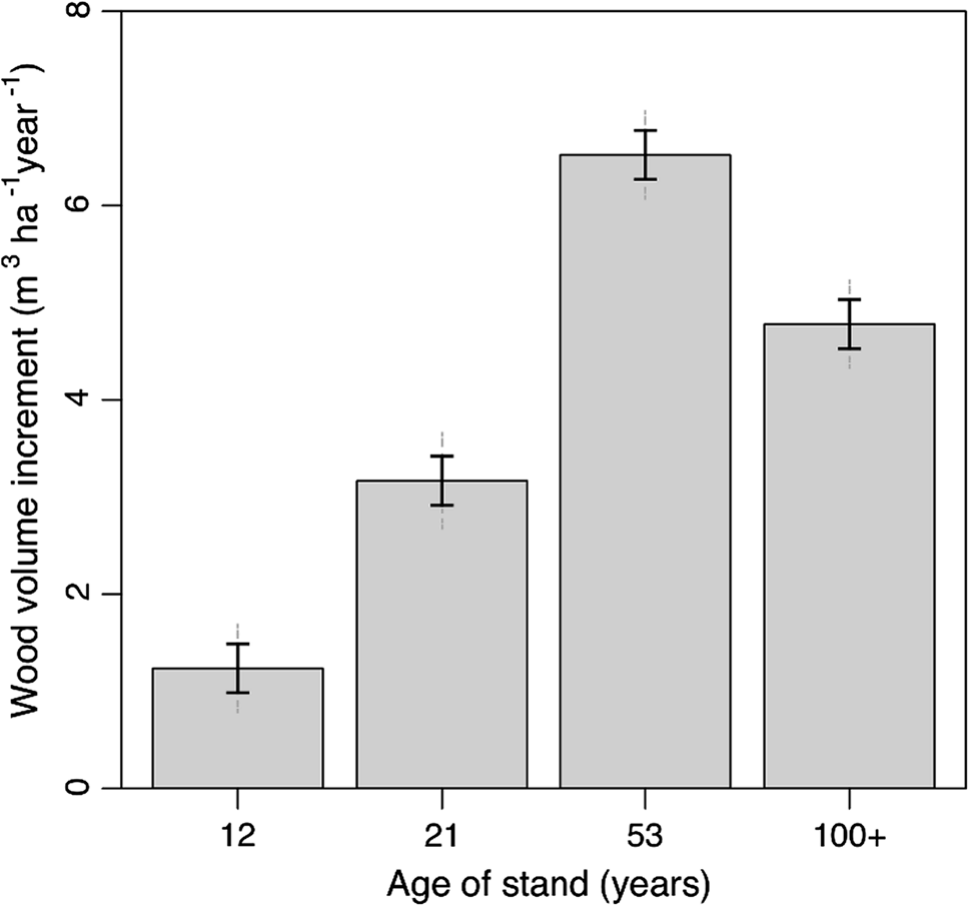
Annual wood volume increment averaged by age class (year of fire), expressed as a yearly average over the previous 5 years (2005–2009). *Solid error bars* represent LSD and *grey dashed error bars* represent 95 % CI (*n* = 4–5). For abbreviations, see Fig. 1

**Fig. 8:**
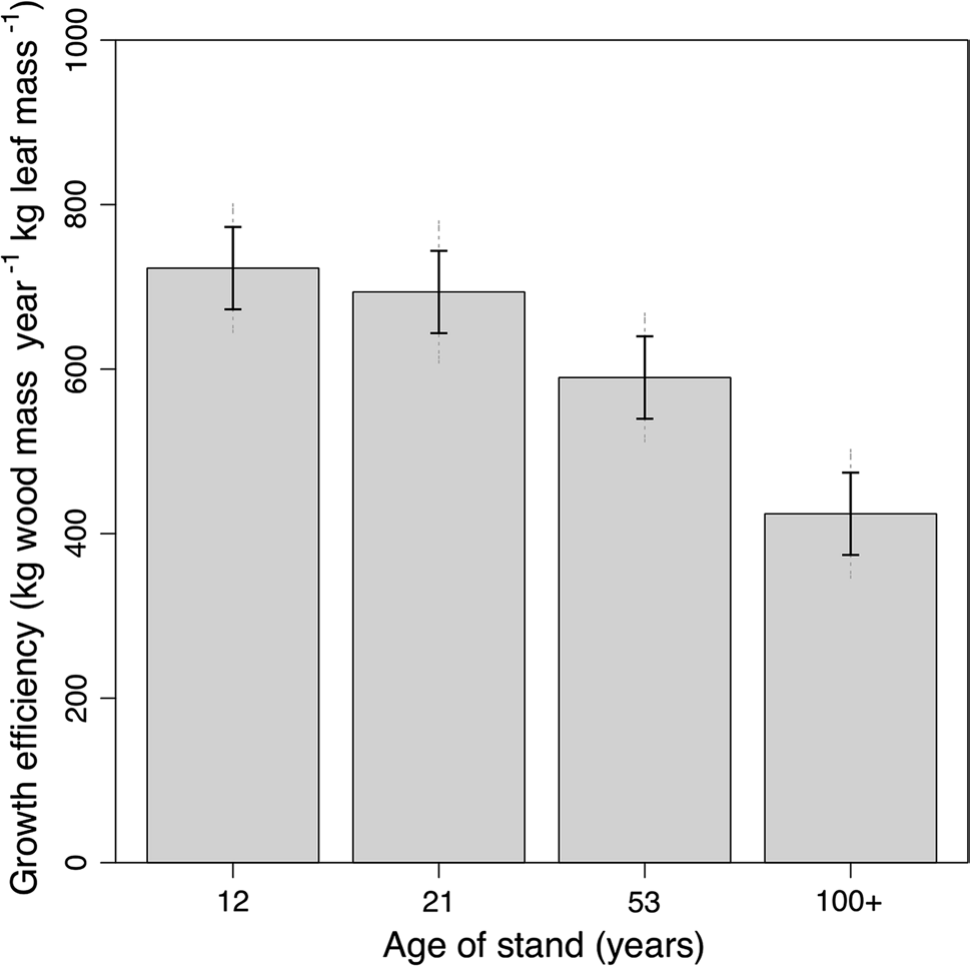
Stand growth efficiency [annual wood volume production or fine root mass (<2 mm) per unit of standing leaf mass] averaged by age class (year of fire). *Error bars* represent 1 SEM (*n* = 4–5)

Mean annual wood volume increment averaged over the 5-year period (2004–2009) doubled from 1.2 m^3^ ha^−1^ year^−1^ in the 12-year-old to 3.2 m^3^ ha^−1^ year^−1^ in the 21-year-old stands (Fig. 7). It then doubled again from the 21- to the 53-year-old stands (Fig. 7). At age 53 years, the mean annual wood volume increment was >6.5 m^3^ ha^−1^ year^−1^ and then declined to 4.8 m^3^ ha^−1^ year^−1^ at age ≥100 years (Fig. 7). Stand growth efficiency, which indicates the production rate of wood relative to existing leaf mass, was highest in the younger stands and consistently declined from 21 to ≥100 years of age (Fig. 8).

## Discussion

We hypothesized that as stands age the fine root (<2-mm diameter) surface area and mass would decline more steeply than leaf area. The present study shows that neither fine roots nor leaf area are declining in the first 100 years post-disturbance in lodgepole pine forests. However, the oldest stands had the highest LAI:fine root area ratio supporting the idea that as stands age tree growth is restricted via limitations of the root system, reducing the potential for soil resource uptake that supplies the leaf area. The continual reduction in stand-level growth efficiency with age further supports a resource-limitation mechanism.

LAI did not peak in the middle age class (53 years old) but instead continued to increase into the ≥100-year-old age class. This result was unexpected and contrasts with early studies in lodgepole pine (Pearson et al. 1984; Smith and Resh 1999); however, these studies had none or very limited site replication within age classes and stocking levels were not consistent (Table 4). In a more recent analysis, with a large pool of sampled sites, Kashian et al. (2013) saw LAI peak around age 40–50 years with decline occurring after age 100 years. The more direct measurement of leaf area that we used in this study avoided the potential underestimation of leaf area which occurs when using light-extinction techniques. This is thought to be a result of the clumped distribution of foliage in crowns, which is not uniform across a chronosequence of stands (Fish et al. 2006).

**Table 4:** Summary of previous studies reporting leaf mass in lodgepole pine (*Pinus contorta*)

	Species	Age (years)	Leaf mass biomass (Mg ha^−1^)	LAI	Tree density (stems ha^−1^)	Basal area (m^2^ ha^−1^)	SDI	No. stands	Foliage biomass methods
Present study	*P. contorta*	12	3.2 (0.6 CI)	2.1 (0.4 CI)	31,831–60,833	8.9–16.2	511–884	5	Allometric equations developed on site
		21	7.4 (0.7 CI)	4.0 (0.4 CI)	11,141–19,298	18.4–34.1	751–1234	5	
		56	11.2 (0.6 CI)	4.7 (0.4 CI)	3378–4807	35.2–51.2	1021–1415	5	
		110	10.8 (0.6 CI)	5.5 (0.4 CI)	1496–2228	42.4–52.2	964–1222	5	
Comeau and Kimmins (1989)	*P. contorta*	70–80	3.9–10.8		1770–3580	–	–	4	Allometric equations developed on site
Pearson et al. (1984)	*P. contorta*	75	12.3		1280	26	632	1	Allometric equations developed on site
		110	8.4–11.4		1850–14,640	42–64	1035–1736	4	
		240	6.9		420	37	671	1	
Litton et al. (2004)	*P. contorta*	10–20	0.2–3.6		425–598,462	0.1–2.2	14–3165	12	Allometric equations developed on site
	*P. contorta*	100-150	6.0 (0.7 SE)		1320–3360	38–52	1043–1115	4	Allometric equations from Comeau and Kimmins (1989)
Smith and Resh (1999)	*P. contorta*	15		1.0	12,500	18.5	759	1	Allometric equations from Long and Smith (1988, 1990, 1992), Smith and Long (1989), Pearson et al. (1984). These studies all took place in the same region
		30		3.8	1075	17.8	451	1	
		50		4.1	1316	23	576	1	
		100		3.3	1766	39.5	942	1	
		260		2.3	1133	42.2	908	1	

Where available, tree density, basal area and SDI (estimated based on available data) are also presented as reference points for comparison

Values* in parentheses* represent either the SEM or 95 % confidence interval (*CI*)

*LAI* Leaf area index; for other abbreviations, see Table 1

There was, however, very little difference in leaf mass between the 53- and ≥100-year-old stands; in fact, the leaf mass in the younger age class was slightly higher. Nevertheless, this pattern contrasts with most other findings that describe leaf area development with time in forest stands (Ryan et al. 1997 for review). However, the absolute measurements of leaf mass and LAI presented in this study are still near the range of variation observed in *P. contorta* stands (Table 4). The reason for the difference in the pattern in leaf area and leaf mass with age is a result of lower specific leaf area (42.1 cm^2^ g^−1^) in the 53-year-old age class compared with the ≥100-year-old age class (50.7 cm^2^ g^−1^). In this region, *P.contorta *stands have been found to have increased levels of empty space between crowns (Fish et al. 2006). Using estimates from Fish et al. (2006), we estimate that crown closure may have declined from 63 to 52 % between the 53- and ≥100-year age classes. This could have also contributed to wider sway patterns and more violent collisions of crowns during wind events (Rudnicki et al. 2003). However, counter to Fish et al. (2006), we observed increasing crown length with stand age (height) in our stands. This may be a unique feature of the stand types chosen for this study, as they were all located on south-facing slopes, thereby allowing illumination of longer sections of crown. The increased foliage clumping (Sampson and Smith 1993; Kucharik et al. 1999; Meng et al. 2006) in stands with high crown shyness would increase self-shading of foliage, thereby explaining the increase in average specific leaf area in the oldest stands.

Fine root surface area increased steadily from 12- to 53-year-old stands with little difference between stands aged 53 to ≥100 years. The same pattern was also observed for fine root mass. Coarse root mass did continue to increase with age, consistent with other *Pinus* studies (King et al. 2007), and likely as a consequence of the greater need for structural support of larger trees. It is plausible that the need for structural coarse root mass diverts energy from the development of fine roots as stands age. Examination of mean annual wood production belowground would support or refute this and would be worth further investigation. Two related root morphological characteristics also declined with age: specific root length (SRL) and specific root area (SRA). In young, vigorously growing stands, root development is prolific, resulting in thinner roots with low wood density (Rosenvald et al. 2013). Thus, older trees are paying higher carbon costs for the fine roots they are producing compared with younger stands. Lastly, shifts in mycorrhizal community composition are known to occur throughout stand development (e.g., Twieg et al. 2007; LeDuc et al. 2013; Rosenvald et al. 2013) and it is possible that these shifts could also be associated with increased carbon demands by the symbionts.

Our study indicates that the older stands had proportionately fewer fine roots to support their leaves. The ratios of LAI to root area and leaf mass to root mass both tended to increase with age (Fig. 6), suggesting that needles in younger stands have potentially greater access to soil resources, such as water, than older stands. Reduced canopy transpiration has been associated with increasing stand age (Delzon and Loustau 2005; Drake et al. 2010). We also observed a clear decline in growth efficiency of stem wood production with age (Fig. 8) further illustrating that leaf area in lodgepole pine is not supporting aboveground wood production to the same degree over time.

Others have found that the relative rate of foliage production scaled with the roots/total belowground allocation across stand ages (Smith and Resh 1999; Hendricks et al. 2006). If this holds true for our study, then a greater proportion of belowground carbon allocation is possibly being diverted to the maintenance and growth of coarse roots. In addition, since root tips are the most distal carbon sink in large trees, they are more likely to be limited in carbon reserves (Landhäusser and Lieffers 2012). Increased construction costs of fine roots with age may also limit the development of new roots (Rosenvald et al. 2013). These factors combined could contribute to the relative decrease in root area/mass observed in the ≥100-year age class.

The lower growing season soil temperatures in the ≥100-year-old stands of 1–3 °C on average (Fig. 2b) are likely the result of a build-up of feather mosses that slowed soil warming in the summer. Colder soils are known to reduce the physiological activity of roots (Tyron and Chapin 1983; Minchin et al. 1994) and therefore reduce the movement of water and nutrients to the stem and leaves. Though not universal (see Ryan et al. 2006 for a review), reductions in stomatal conductance have been observed with stand age (Drake et al. 2010). Reduced physiological activity of roots may have reduced the sink strength, thereby reducing movement of carbon to roots. Further, cold soils may reduce the mineralization rate, explaining the decline in available phosphorus and potassium at the forest floor-mineral soil interface in the ≥100-year-old stands compared with the 53-year-old stands (Fig. 1). However, total nitrogen in foliage was similar across the three older age classes (Fig. 5c) corresponding with similar values in soil available inorganic nitrogen (Fig. 1) and contrasting with Olsson et al. (1998) where inorganic soil nitrogen declined between 30 and 100 years of age.

### Soundness of root sampling methodology

We are confident in our estimates of fine root surface area based on the following reasoning. Firstly, the quantity of fine roots sampled far exceeded the soil volume sampled in previous studies (Xiao et al. 2003; Ruess et al. 1996; Litton et al. 2003). Secondly, estimates of fine root mass are within the ranges reported in previous studies of *Pinus* species (Table 5). Thirdly, we accounted for loss of roots during the sampling process. During field sampling, we initially sieved soil samples and picked out root fragments and pieces; this “missed” fraction contributed, on average, 3 % to the total fine root mass. In addition, during the soil core washing stage in the laboratory, small root tips and fragments (1- to 3-mm length) tended to be washed out in the washing process (organic pool). Therefore, we collected and sub-sampled from this organic pool and hand-sorted any visible root fragments (Teste et al. 2012) which contributed, on average, 50 % to the total fine root mass pool. Coarse root estimates in the present study are representative of lateral root structure only, as it is likely that sampling underestimated the coarse root contribution; this is because inclusion of stumps, which can contribute 50–80 % of coarse root biomass (Ostonen et al. 2005; King et al. 2007), was not practical in our sampling design.

**Table 5:** Summary of previous studies reporting fine and coarse root mass for various *Pinus* species

	Species	Age (years)	Density (stems ha^−1^)	Fine root biomass (Mg ha^−1^)	Total root biomass (Mg ha^−1^)	No. stands	Fine root biomass methods	Coarse root biomass methods
Present study^a^	*Pinus contorta*	12	31,831–60,833	2.2 (0.8 CI)	2.2 (0.3 CI)	5	Soil cores (square cores 17–43 cm)	
		21	11,141-19,298	4.0 (0.8 CI)	6 (0.4 CI)	4		
		56	3378–4807	5.1 (0.8 CI)	10 (0.3 CI)	5		
		110	1496–2228	4.9 (0.8 CI)	12 (0.3 CI)	5		
Comeau and Kimmins (1989)^b^	*P. contorta*	70–80	1770–3580	4.3–6.4	30–73	4	Soil cores [5-cm (forest floor) and 10-cm diameter (mineral soil)]	Allometric equations developed on site
Litton et al. (2003)	*P. contorta*	10–20	425–598,462	0.2–1.8	0.3–3.6	12	Soil cores (6.35-cm diameter)	Allometric equations developed on site
Pearson et al. (1984)	*P. contorta*	75	1280	n.r.	26	1	–	Plane-intersect method for root determination, scaled by basal area
		110	1850–14,640	n.r.	38–56	4	–	
		240	420	n.r.	38	1	–	
Litton et al. (2004)	*P. contorta*	100–150	1320–3360	1.4 (0.1 SE)	21 (3.6 SE)	4	Allometric equations from Comeau and Kimmins (1989)	
Xiao et al. (2003)	*Pinus sylvestris*	70–80	n.r.	2.2	19.2	1	Soil cores [15-cm (forest floor) and 8-cm diameter (mineral soil)]	Allometric equations developed on site
King et al. (2007)^a^	*Pinus resinosa*	2–8	1750–2400	n.r.	0.06–2.1	4	–	10 × 15-m, 10 × 10-m plot; complete excavation
		12–17	1750	n.r.	5.6–8.5	2	–	15 × 15-m plot; complete excavation
		20–35	1750–2400	n.r.	3.6–23.6	2	–	
		55	622	n.r.	23.3	1	–	

Values * in parentheses* represent either SEM or 95 % CI

*n.r.* Not recorded

^a^Total root biomass does not include stump

^b^Fine root biomass was <5-mm diameter

### Conclusion

As expected, we observed a clear decline in annual wood volume increment from age 53–≥100 years. However, this did not correspond with a decline in leaf area (which actually increased) or fine root area. Declining growth efficiency, even before stand-level decline in annual wood volume increment, indicates that these stands were not effectively using their leaf area. Lower relative quantities of fine roots as well as colder soils were likely contributors to declining growth with age. These results indicate that, as stands age, belowground processes will likely play a greater role in the performance of these forests, potentially increasing their susceptibility and vulnerability to stress, ultimately leading to a higher risk of mortality.

## Electronic supplementary material

Below is the link to the electronic supplementary material.

Supplementary material 1 (DOCX 2299 kb)
