# Supplementary material for: Viewing forests from below: fine root mass declines relative to leaf area in aging lodgepole pine stands
Source: Oecologia. 2016 Apr 4;181:733–47. doi: 10.1007/s00442-016-3621-6 (PMC4912597; doi:10.1007/s00442-016-3621-6)
Supplement: Supplementary file 1 — Supplementary material 1 (DOCX 2299 kb) [file 442_2016_3621_MOESM1_ESM.docx]

**Online Resource A:** Site and tree characteristics

Figure A1: Examples of *Pinus contorta* study sites by age class: (a) 12 year old, (b) 20 year old, (c) 53 year old and (d) 100+ year old stands.


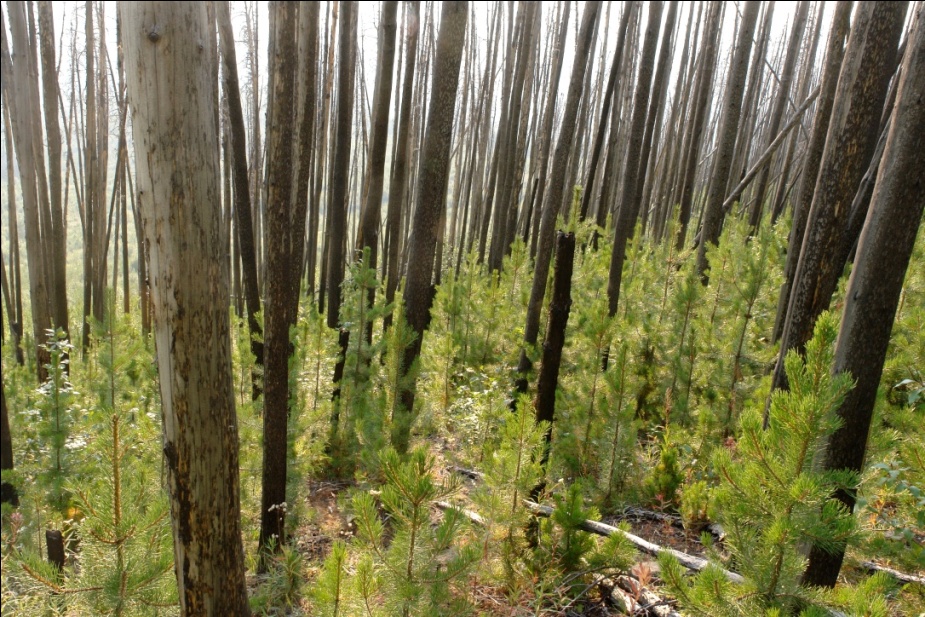

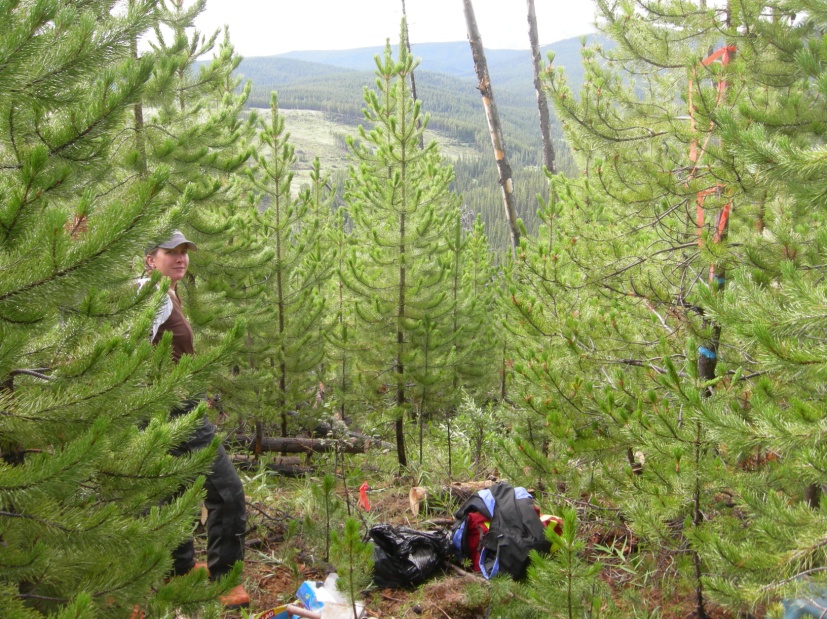


(b)

(a)


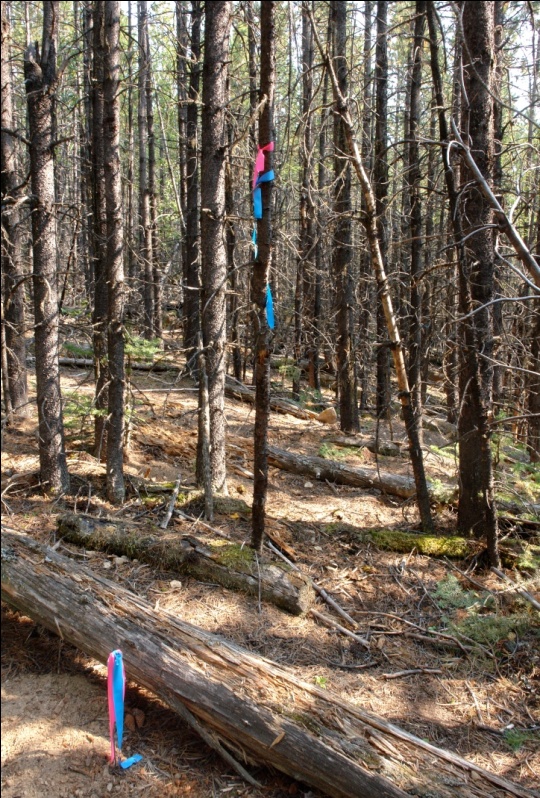

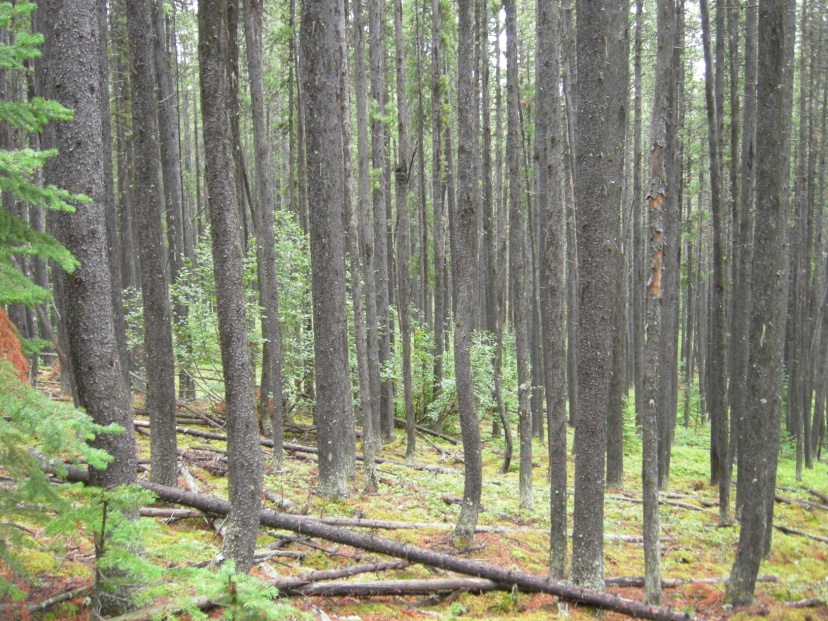


(d)

(c)

**Online Resource B –** Root sampling and washing procedure

Field sampling:

The upper portion of the sample was typically a large ‘mat’ of understory shrub, grass and pine roots and organic material in which case it could not be sifted and was placed directly into plastic bags. The mineral soil portions of each layer were initially sieved (4 mm mesh) in the field in order to reduce the soil to be carried out. Care was taken to minimize disturbance of intact root mats (combination of coarse and fine roots). In order to account for loss of roots from coarse sieving in the field, a sub-sample of the soil that passed the sieve was additionally collected and brought back to the lab. Fine roots were manually separated (see also methodology below) and the estimate of the fine roots lost by coarse sieving (hereafter called the field fine root fraction, FFRF) was scaled up to the subsample-level:

$FFRF= \frac{mass fine roots collected}{\frac{mass soil collected}{soil bulk density}}\times volume of soil sieved$

Soil bulk density samples were collected concurrently with root samples at 15 cm depth using a stainless steel circular core (5 cm diameter x 5 cm depth). Samples were collected in plastic bags and stored until lab processing. Soil bulk density (g cm^-3^) samples were oven-dried at 105°C until weight constancy. Bulk density was determined as:

$\mathrm{bulk} density=\frac{dry soil mass}{soil core volume}$

Laboratory root washing procedure:

The root washing and separation procedure followed Teste, Lieffers & Strelkov (2012). Soil samples were dry-sieved (4mm and 1.4 mm) and then were sieved wet (1.0mm, 0.8mm and 250 μm) to separate roots from soil. Even with washing, in the upper 15 cm there would remain a dense mat of moss fragments, shrub roots and in some stands, grass roots. These samples would then be immersed in water and lodgepole pine roots were manually removed with tweezers (hereafter called the lab fine root fraction, LFRF). Lodgepole pine roots were easily differentiated by distinct coloration and structure relative to roots of other species (grasses and shrubs). Live roots were separated from dead roots based on color and texture (smooth and plump were indicative of live roots while granular and withered were indicative of dead or dying roots) of the cortex (Ruess et al. 1996) and the flexibility of the roots (e.g: did not crumble or further fragment when handled with tweezers) (Comeau & Kimmins 1989). Coarse roots (>2mm in diameter) were separated from fine roots (hereafter lab coarse root fraction, LCRF). Separated pine roots were placed in plastic bags and frozen at -18°C until determination of surface area and diameter.

Small root fragments that had broken off the larger root clusters during washing were collected in a 250 μm sieve. At this sieve size materials would include root fragments, organic matter and woody debris. This material was hand shaken in enough water to create a thick slurry and approximately 5 mL sub-sampled. In practice, the subsample represented 0.06-2.4% of the total dry mass of the organic fraction collected in the 250 sieve and the actual quantity of fine roots constituted on average 10-25% of the organic fraction. The subsample was placed in a separate tray of water and pine root fragments were manually separated from remaining organic matter (hereafter called the fine root particulate fraction, FRPF). Both the larger particulate slurry and separated, sub-sampled particulate fraction were also frozen until subsequent analysis.

$fine root density \left( \mathrm{kg}m^{-2}\mathrm{or}m^{2}m^{-2} \right)=\frac{FFRF+LFRF+FRPF}{total soil sampled area}$

**Online Resource C:** Age-specific stand leaf area sampling and estimation

Stand leaf area was estimated by a series of regression models for each stand age category. First in each plot two trees were selected in order to represent the span of possible tree sizes/classes (e.g. dominant, co-dominant or suppressed across the five replicates). These trees were felled and the height and diameter at 1.3 m were measured. On each, the crown was separated into 1 m sections (53 and 100+ year old stands) or 0.5m sections (12 and 21 year old stands). Within each section, the branches were removed and the length and base diameter measured. Three branches (spanning a range of sizes) from each section (12-21 branches per tree) were subsequently collected and brought to the lab for scaling branch leaf mass and area. Along the length of the entire crown, sub-samples of needles of all ages were collected from each tree in order to determine a relationship between leaf-area and leaf-mass. These needles were collected in a plastic bag and frozen (within 8 hours of harvest) until further processing.

Sampled branches were placed in paper bags at room temperature and then dried at 70°C for one week. Needles were separated from stems and weighed. Linear regression was used to predict branch leaf mass using branch diameter and length diameter as predictors, within each of the four lodgepole pine age classes. All regression models were analyzed using R software (R Core Team 2014) and fitted using the LMER function (R package Lme4). Eleven candidate models with parameters for branch length, diameter, crown section and/or a random effect for the individual tree were generated and compared (Online Resource, Table E1). The models with greater than 1% probability (Online Resource, Table E2) were used in model averaged estimates (model parameter estimates shown in Online Resource, Table E3). Model-averaging was a weighted average based on model probabilities (Anderson 2008) which are given as:

$\mathrm{Delta}_{(i)}=\mathrm{AIC}_{(i)}-\mathrm{AIC}_{(model smallest AIC)}$

$\mathrm{Probability}_{(i)}=\frac{\mathrm{Likelihood}_{(i)}}{\sum_{1}^{11} \mathrm{Likelihood}}-\mathrm{Likelihood}_{(i)}=e^{-0.5\times\mathrm{Delta}_{(i)}}$

This allowed for estimation of the leaf mass of each branch measured on the 38 felled trees above. From these estimates, whole tree leaf mass was calculated.

Using the tree leaf mass estimates for the 38 trees above, a similar approach was used to generate predictive models of whole tree leaf mass from parameters including: height, diameter, crown length and stand age. Eleven candidate models (Online Resource, Table E4) within each age class were generated and compared. The best models were used to generate model-averaged (weighted by the proportion of AIC deviance from the best model) predictive estimates of tree leaf mass of all individual trees (Online Resource, Table E5) within each plot (Online Resource, Table E6). The leaf mass of each tree was then converted to leaf area with the specific leaf mass estimates described below.

Sub-sampled needles, which were stored frozen, were scanned with a flatbed scanner and one-sided leaf area measured with WinFolia software (Regent Instruments Inc.). Needles were oven-dried at 70°C for two days and weighed. Specific leaf mass was calculated for each tree and averaged for each age class (Table 1). This ratio was used to estimate tree leaf area from tree leaf mass. Leaf area index (LAI) for each stand is given as:

$\mathrm{LAI}_{(stand (i))}=\frac{\sum_{1}^{n} tree leaf area (m^{2})}{plot area (m^{2})}$ (8)

**Online Resource D:** Prediction of 2004 diameter and mean annual volume increment

Three candidate models (Online Resource, Table E7) were compared for the prediction of diameter in 2004 within each age class. All regression models were analyzed using R software (R Core Team 2014) and fitted using the LM and/or LMER function (R package Lme4). The model selection of the lowest AIC model (Online Resource, Table E8) was used to generate estimates of 2004 diameter of trees within measurement plots (Online Resource, Table E9). Similarly, models for prediction of height, which were required to correspond with estimates of 2004 diameter, were also generated from plot data (with height and diameter) obtained in 2009. These candidate models had the same structure as described above (Online Resource, Table E7) and the model with the lowest AIC (Online Resource, Table E10) was used for prediction 2004 heights (Online Resource, Table E11).

Tree volume was determined from estimated diameter and tree heights, for each tree in the stand for the years 2004 and 2009. For the younger age classes (12 and 21 years old), the volume of a cone was used as a proxy for tree volume. For the older stands (53 and 100+ years old), we utilized taper equations specific to lodgepole pine from the upper foothills of Alberta (Huang 1994). Detailed descriptions of taper equations are found in Online Resource F. Mean annual volume increment was determined as:

$Mean annual volume increment \left( m^{3}\mathrm{year}^{-1} \right)=\frac{2009 volume-2004 volume}{5}$

**Online Resource E:** Tables (1-11) summarizing equations used for estimation of leaf mass and wood volume production.

**Table E1** Candidate regression models used to predict branch leaf mass. Individual trees had 12-21 branches sampled. Regression models were generated for each of the four lodgepole pine age classes (12, 21, 53 and 100+ years).

**Table E2** AIC results from linear regression of branch leaf mass. Values in bold are models that were used to average biomass estimates (see also Table B3). Delta values were calculated as the relative difference between the model with lowest AIC and model AIC.

**Table E3** Branch-level leaf mass prediction equations for all age classes.

**Table E4** Candidate regression models used to predict tree-level leaf mass. Two trees were sampled from each site (n=10 for each age class). Regression models were generated for each of the four lodgepole pine age classes (12, 21, 53 and 100+ years).

**Table E5**  AIC results from linear regression of tree leaf mass (Table B4). Values in bold are models that were used to average biomass estimates. Delta values were calculated as the relative difference between the model with lowest AIC and model AIC.

**Table E6**  Model parameters for tree level model of leaf mass prediction.

**Table E7**  The observed diameter in 2009 was used to generate predictive models of previous year’s diameters (2004) and height. Candidate regression models used to predict tree-level leaf mass. Two trees were sampled from each site (n=10 for each age class). Regression models were generated for each of the four lodgepole pine age classes (12, 21, 53 and 100+ years).

**Table E8** Summary of AIC scores from linear regression models for all predictions of 2004 diameter (models from Table B7). AIC score in BOLD was used.

**Table E9** Results from linear regression of 2004 diameter against observed 2009 diameter. These models were used to predict diameter of trees in 2004.

**Table E10**  Summary of AIC scores from linear regression models (Table C9). AIC score in BOLD was used for all predictions of height from diameter.

**Table E11**  Results from linear regression used to determine tree height (from diameter) for 2004 and 2009 volume estimation.

**Online Resource F:** Tree volume determination

Determination of tree volume follows Huang et al. (1994) and is described as:

$$d={a_{0}D}^{a1}a_{2}^{D}X^{y*}$$

where:

$$y^{*}=b_{1}Z^{2}+b_{2}\ln\left( Z+0.001 \right)+b_{3}\sqrt{Z}+b_{4}e^{Z}+b_{5}(\frac{D}{H})$$

$$X=(1-\sqrt{h/H}/(1-\sqrt{p})$$

and:

*d* = diameter inside bark at h (cm)

*h* = height above the ground (m)

*H* = total tree height (m)

*D* = diameter at breast height outside bark (cm)

*Z* = h/H

*p* = location of the inflection point, assumed to be at 22.5% of total height above the ground.

e = base of the natural logarithm

The following coefficients are from Huang (1994) and are specific to lodgepole pine in the upper foothills and subarctic ecological areas of Alberta:

a_0_ = 0.828665

a_1_ = 1.024196

a_2_ = 0.997492

b_1_ = 0.596193

b_2_ = -0.118777

b_3_ = 0.465591

b_4_ = -0.196176

b_5_ = 0.083094

Then the diameter of the inside of the bark is required based on known diameter of the outside bark:

$$DOB=a+bDIB$$

DOB = diameter outside bark (cm)

DIB = corresponding diameter inside the bark (cm)

As above, coefficients specific to lodgepole pine in upper foothills and sub-arctic ecological areas:

a = 0.308258

b = 1.024549

Then, the diameter of the stump is estimated, based on known diameter at breast height:

$${DOB}_{stp}=a+bD+cD^{2}$$

DOB_stp_ = stump diameter outside bark (cm)

Coefficents specific to lodgepole pine in upper foothills and sub-arctic ecological areas:

a = -0.487166

b = 1.112282

c = 0.000347

**Online Resource G:** Soil available macro and micronutrients from PRS resin probes.

**Figure G1:** Soil available macronutrients recovered from PRS resin probes (5 probes pooled in each stand into a single sample) averaged by age class (year of fire). (a) total N (nitrate and ammonium), (b) nitrate, (c) ammonium, (d) phosphorus, (e) potassium, (f) calcium and (g) magnesium. Means to the left of the vertical line were resin probes inserted at a 45° angle into the mineral soil. Means to the right of the vertical line were resin probes inserted horizontally at the forest floor-mineral soil interface. This could only be accomplished in the older age classes as the younger age classes lacked forest floor development. Solid error bars represent least-significant difference intervals and dotted error bars represent 95% CI (n=4-5).

**Figure G2:** Soil micronutrients recovered from PRS resin probes (5 probes pooled in each stand into a single sample) averaged by age class (year of fire). (a) iron, (b) manganese, (c) zinc, (d) boron, (e) sulfur, (f) aluminum. Means to the left of the vertical line represent resin probes inserted at a 45° angle into the mineral soil. Means to the right of the vertical line represent resin probes inserted horizontally at the forest floor-mineral soil interface. This could only be accomplished in the older age classes as the younger age classes lacked forest floor development. Solid error bars represent least-significant difference intervals and dotted error bars represent 95% CI (n=4-5).
